# MambaSSM: efficient segmentation of brain structures in anisotropic 3D EM images via state-space models

**DOI:** 10.3389/fnins.2026.1772632

**Published:** 2026-04-16

**Authors:** Minjie Liu, Laifu Fang, Qinhu Zhang, Hongyu Yang

**Affiliations:** 1 Department of Obstetrics, The Affiliated People's Hospital of Ningbo University, Ningbo, China; 2 Department of Pathology, The Affiliated People's Hospital of Ningbo University, Ningbo, China; 3 Ningbo Institute of Digital Twin, Eastern Institute of Technology, Ningbo, China

**Keywords:** anisotropic 3D image, brain structure, electron microscopy, instance segmentation, Mamba, state-space model

## Abstract

Accurate segmentation of brain structures from anisotropic 3D electron microscopy (EM) images remains challenging due to the trade-off between global context modeling and computational efficiency. While state-space models (SSMs) like Mamba have shown promise in capturing long-range dependencies, their direct application to anisotropic EM data has been limited. We introduce MambaSSM, a novel network that adapts SSMs to anisotropic 3D EM images via a tailored scanning strategy. Our method features two core modules: an SSM-based anisotropic adaptation module for early-stage feature learning and an SSM-based isotropic adaptation module for later-stage refinement. These modules are interleaved with convolutional layers to enable multi-scale feature extraction. Evaluated on two public datasets (SNEMI3D and MitoEM-R), MambaSSM achieves superior segmentation accuracy with significantly lower memory usage compared to CNN, Transformer, and Mamba based baselines.

## 1 Introduction

Brain structure refers to the anatomical organization of the brain, encompassing various tissues, regions, and cellular components such as neurons and their synaptic connections. Reconstruction of these neural structures at the cellular level is essential for advancing our understanding of neural connectivity and brain functions (Liu et al., 2024b; Li et al., 2021; Mai et al., 2023).

In recent years, serial electron microscopy (EM) has been widely adopted in neuroscience research due to its nanometer-resolution imaging capabilities, enabling detailed visualization and precise analysis of neural ultrastructures, including synapses, organelles, and neuronal processes (Zheng et al., 2018; Harris et al., 2006; Knott et al., 2008; Tapia et al., 2012). The primary goal of EM image analysis is to accurately segment and reconstruct these fine structures, which serves as the foundation for subsequent connectivity analysis and functional studies. To achieve this, many studies (Li et al., 2022; Lee et al., 2017; Çiçek et al., 2016) have employed convolutional neural networks (CNNs) to extract hierarchical features from EM images and perform pixel-wise classification for structure segmentation. A significant factor contributing to performance improvements in these models is the expansion of the network's contextual aggregation range through deeper architectures and larger kernels. A broader spatial context allows the model to incorporate more surrounding information, facilitating more accurate identification of boundary pixels and improving segmentation consistency (Luo et al., 2016). Furthermore, enhanced contextual modeling enables the network to capture hierarchical and abstract features, which is essential for distinguishing subtle morphological differences in EM image segmentation (Guo et al., 2024).

Recently, the vision transformer (ViT) framework (Dosovitskiy, 2020), which adapts the self-attention mechanism (Vaswani et al., 2017) from natural language processing to capture global spatial information, has gained significant traction in 3D biomedical image segmentation tasks. However, the high dimensionality introduced by the 3D nature of biomedical data imposes significant computational and memory demands, particularly for self-attention operations that scale quadratically with input size, thereby hindering the efficiency and practicality of transformer-based approaches for high-resolution EM volumes (Xing et al., 2024). To address the challenges of long-sequence modeling in high-dimensional data, Mamba (Gu and Dao, 2023), inspired by state-space models (Gu et al., 2021), introduces novel linear-time scanning strategies to efficiently capture long-range dependencies. By integrating selective state-space mechanisms and hardware-aware parallel algorithms, Mamba improved computational efficiency while maintaining global context modeling, making it particularly well-suited for high-dimensional biomedical data processing (Gu and Dao, 2023; Xing et al., 2024).

However, due to the physical constraints of serial sectioning and imaging setups, 3D EM images typically exhibit anisotropic voxels (Wei et al., 2020; Zeng et al., 2017), meaning that the spatial resolution in the xy plane (perpendicular to the cutting axis) is substantially higher than along the z dimension (the cutting axis). Specifically, while the z-resolution remains constant throughout the imaged volume, it is often 5–10 times lower than the xy-resolution. This anisotropy creates distinct feature scales across dimensions that standard isotropic processing cannot adequately handle. Previous approaches have addressed this challenge through various strategies, including anisotropic network architectures (Lee et al., 2017), multi-scale feature fusion (Li et al., 2022), and specialized loss functions (Zeng et al., 2017), yet these methods remain limited by the localized nature of CNN operations. Although vision transformers have been explored for 3D medical image segmentation (Hatamizadeh et al., 2022), their direct application to anisotropic EM data is computationally prohibitive and fails to exploit the inherent resolution asymmetry.

To address these challenges, we introduce a novel network that adapts Mamba (Gu and Dao, 2023) for anisotropic 3D EM image segmentation. Specifically, we consider the anisotropic voxel properties and propose a directional scanning strategy that respects the resolution disparity between xy and z dimensions to extract informative features from anisotropic EM volumes. Building on this scanning scheme, we develop a state-space model SSM-based anisotropic adaptation module to enhance feature learning under anisotropic conditions. In addition, to facilitate multiscale feature extraction, we interweave the SSM-based anisotropic adaptation module with convolutional down-sampling layers. As down-sampling progresses, the effective resolution ratio between dimensions gradually decreases, making the feature representations approximately isotropic. To accommodate this transition, we propose a secondary scanning strategy optimized for isotropic adaptation. Building on this approach, we introduce an SSM-based isotropic adaptation module to refine feature learning in nearly isotropic representations. Similarly, convolutional down-sampling layers are interwoven with the SSM-based isotropic adaptation module to support hierarchical multi-scale feature learning.

In summary, our key contributions are as follows:

- We address the anisotropic nature of 3D EM images by proposing a targeted scanning strategy that effectively captures global information.
- Building on this strategy, we develop an SSM-based anisotropic adaptation module and an SSM-based isotropic adaptation module, which form the core of our network. By interleaving convolutional layers with these modules, we achieve robust multi-scale feature learning.
- We validate our approach through extensive experiments on two 3D EM image segmentation datasets, demonstrating its superior performance and low memory consumption compared to existing methods.

## 2 Related work

**Figure 1:**
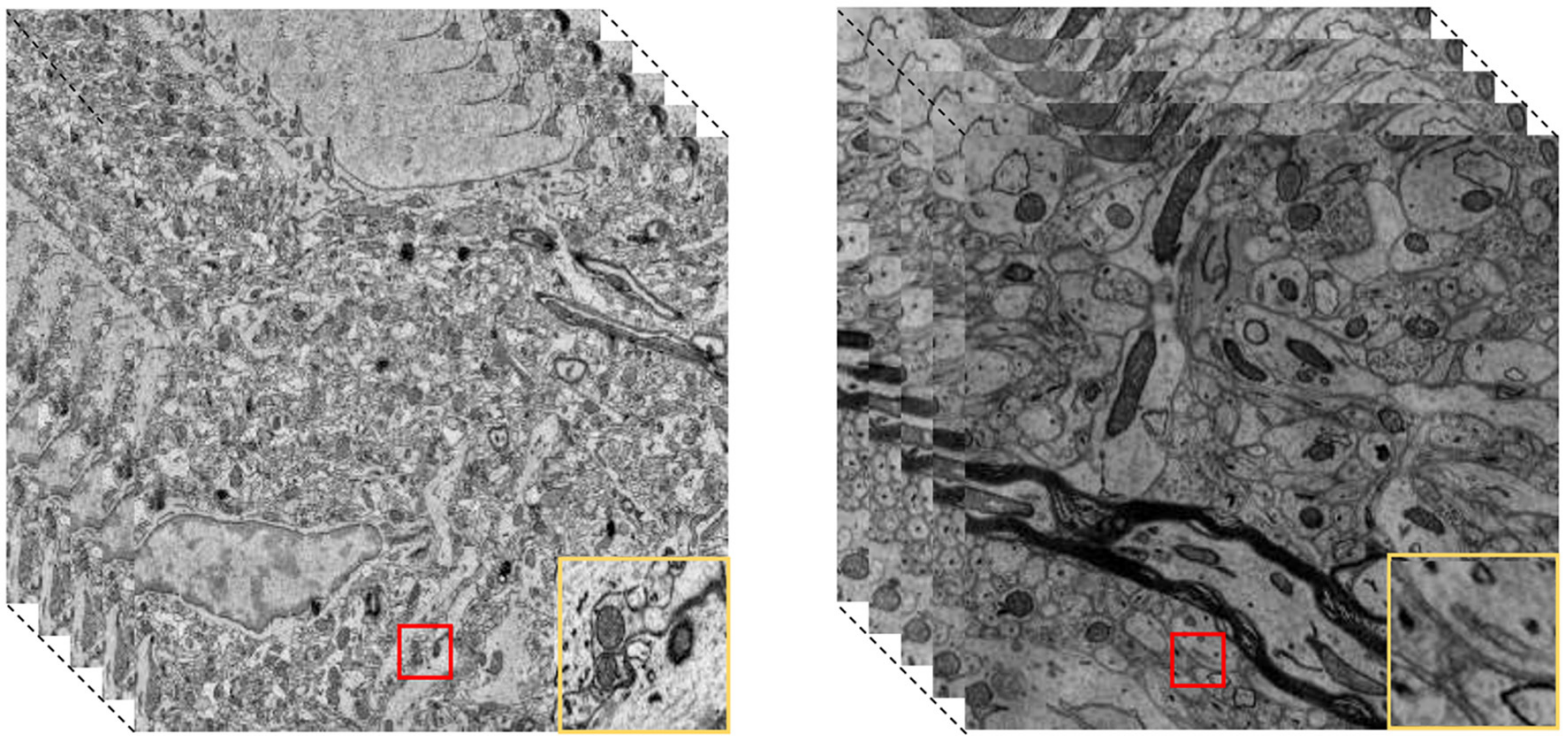
Brain structure in 3D EM images. The red box contains the brain structure, and the yellow box contains the details of the brain structure.

Electron microscopy is a fundamental tool for advancing neuroscience research, enabling the visualization of cellular and subcellular structures with nanometer precision (Zeng et al., 2017). As shown in the Figure 1, through precise segmentation of 3D EM images, specific neural tissues—including neurons, synapses, and organelles such as mitochondria—can be effectively reconstructed (Wei et al., 2020), offering critical insights into underlying biological functions and disease mechanisms (Zeng et al., 2017; Kasthuri et al., 2015). With the rapid development of deep learning, data-driven segmentation methods have gradually become mainstream solutions. Among these, convolutional neural network (CNN)-based and transformer-based architectures have been widely adopted for volumetric biomedical image segmentation. CNN-based methods, such as 3D U-Net (Çiçek et al., 2016), use 3D convolution operations for accurate local feature extraction and have become one of the most commonly used deep learning architectures in this domain. However, due to the localized nature of convolution operations, CNN-based models struggle to capture long-range spatial dependencies in large-scale image volumes, which limits their effectiveness when processing structures with extensive contextual relationships.

In contrast, transformer-based methods have shown unique advantages in global context modeling. Leveraging the self-attention mechanism (Vaswani et al., 2017), these methods can effectively capture long-range dependencies, thereby addressing a key limitation of traditional CNN methods. For example, the UNETR (Hatamizadeh et al., 2022) model uses a vision transformer (ViT) as an encoder to learn global context information and integrates it with a CNN-based decoder through multi-scale skip connections, significantly improving segmentation accuracy. Similarly, SwinUNETR (Hatamizadeh et al., 2021) employs Swin Transformer (Liu et al., 2021) as an encoder to extract hierarchical multi-scale features, coupled with a carefully designed decoder to fuse features from different stages, achieving strong performance on volumetric biomedical image segmentation tasks. However, the self-attention mechanism (Vaswani et al., 2017), while effective for global modeling, introduces substantial computational overhead. The quadratic complexity of self-attention with respect to sequence length results in high memory consumption and slow inference speeds, which limits the practical applicability of these methods for high-resolution 3D EM data.

State-space models (SSMs) (Gu and Dao, 2023; Smith et al., 2022; Gu et al., 2021) originate from classical control theory and have emerged as a powerful framework for sequence modeling in deep learning. A key characteristic of SSMs is their ability to model long-range dependencies with linear computational complexity relative to sequence length, offering significant advantages for processing long sequences. Recently, Mamba (Gu and Dao, 2023), a data-dependent SSM incorporating a selective mechanism and hardware-aware optimization, has demonstrated superior performance over transformers in natural language processing tasks while maintaining linear scaling with input length. Building on the success of transformers in computer vision, researchers have begun adapting Mamba for visual tasks, including biomedical image analysis (Liu et al., 2024a; Shi et al., 2024; Xing et al., 2024). For instance, SegMamba (Xing et al., 2024) enhances 3D feature modeling by designing a three-way Mamba module that processes spatial information from three orthogonal directions, effectively capturing long-range dependencies in volumetric data while maintaining computational efficiency.

However, many 3D EM images exhibit anisotropic voxels, where the spatial resolution differs significantly across dimensions, posing unique challenges that limit the direct application of the aforementioned architectures. Several studies have proposed specialized solutions to address anisotropy in 3D EM images. Superhuman (Lee et al., 2017) introduces three specific design choices to better handle the high anisotropy of serial section electron microscopy images: anisotropic convolution kernels, multi-scale feature fusion, and specialized training strategies. Res-UNet-R (Li et al., 2022) designs an anisotropic convolution block consisting of a 1 × 3 × 3 convolutional layer followed by two cascaded 3 × 3 × 3 layers with inserted skip connections, aiming to gradually expand the contextual range while respecting dimensional asymmetry. To better adapt to anisotropic resolution in serial section EM images, U3D-BC (Wei et al., 2020) modifies the standard U-Net (Ronneberger et al., 2015) architecture by initially employing asymmetric 3D convolutions and transitioning to symmetric 3D convolutions after down-sampling reduces the effective anisotropy ratio.

These methods demonstrate that careful architectural design can improve the processing of anisotropic 3D EM images. However, their reliance on convolutional operations fundamentally limits their ability to capture long-range dependencies, particularly along the high-resolution lateral dimensions where extensive contextual information is crucial for accurate segmentation.

## 3 Methodology

### 3.1 State space model

Structured State Space Sequence Models (S4) (Gu et al., 2021) and Mamba draw inspiration from continuous dynamical systems, where in a one-dimensional input sequence *x*(*t*)∈ℝ is transformed into an output sequence *y*(*t*)∈ℝ through the intermediary of a hidden state *h*(*t*)∈ℝ^*N*^. In this framework, the evolution of the system is governed by the parameter matrix **A**∈ℝ^*N*×*N*^, while the input and output projections are parameterized by **B**∈ℝ^*N*×1^ and **C**∈ℝ^1 × *N*^, respectively.


$$\begin{array}{l} h^{\prime }\left(t\right)=\text{A}h\left(t\right)+\text{B}x\left(t\right),\\ y\left(t\right)=\text{C}h\left(t\right). \end{array}$$ (1)

The S4 and Mamba are the discrete versions of the continuous system, which include a time scale parameter **Δ** to transform the continuous parameters **A**, **B** to discrete parameters $\bar{\text{A}},\bar{\text{B}}$. Various discretization rules can be used, a fixed formula is $\bar{\text{A}}=f_{\text{A}}\left(\Delta ,\text{A}\right)$ and $\bar{\text{B}}=f_{\text{B}}\left(\Delta ,\text{B}\right)$, where the pair (*f*_**A**_, *f*_**B**_) is called a discretization rule. The commonly used method for transformation is zero-order hold (**ZOH**), which is defined in Equation 2:


$$\begin{array}{l} \bar{\text{A}}=\exp\left(\Delta \text{A}\right),\\ \bar{\text{B}}={\left(\Delta \text{A}\right)}^{-1}\left(\exp\left(\Delta \text{A}\right)-\text{I}\right)\cdot \Delta \text{B}. \end{array}$$ (2)

After the discretization of **A**, **B**, the discretized version of Equation 1 using a step size **Δ** can be rewritten in Equation 3:


$$\begin{array}{l} h\left(t\right)=\bar{\text{A}}h_{t-1}+\bar{\text{B}}x\left(t\right),\\ y\left(t\right)=\text{C}h\left(t\right). \end{array}$$ (3)

At last, the models compute output through a global convolution, as in Equation 4.


$$\begin{array}{l} \bar{\text{K}}=\left(\text{C}\bar{\text{B}},\text{C}\bar{\text{A}}\bar{\text{B}},\ldots ,\text{C}{\bar{\text{A}}}^{M-1}\bar{\text{B}}\right),\\ \text{y}=\text{x}*\bar{\text{K}}, \end{array}$$ (4)

where M is the length of the input sequence *x*, and $\bar{\text{K}}\in {\mathbb{R}}^{M}$is a structured convolutional kernel.

### 3.2 Residual vision state-space module

**Figure 2:**
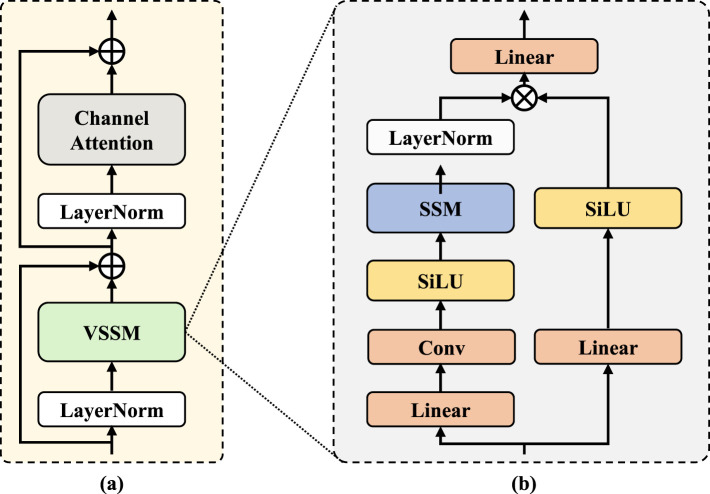
**(a)** The detailed structure of Residual Visual State-Space Module (RVSSM). The RVSSM follows a typical transformer-style design. **(b)** The detailed structure of Visual State Space Module (VSSM).

The Visual State-Space Module (VSSM) (Liu et al., 2024c) is designed based on a state-space model, enabling the capture of long-range dependencies. The architecture of VSSM is shown in Figure 2. The design of the transformer module mainly follows the following sequence: Norm → Attention → Norm → MLP. Additionally, as discussed in Guo et al. (2024), the SSM typically introduces a large number of hidden states to memorize long-range dependencies, leading to significant channel redundancy. Therefore, to allow the SSM to focus on learning distinct channel representations, we introduce a channel attention mechanism (Hu et al., 2018) which allows the SSM to focus on selecting the most relevant channels through subsequent channel-wise attention. Based on the above analysis, we design the residual visual state-space module(RVSSM). The RVSSM is shown in Figure 2a. Given an input deep feature *Z*^*l*^∈ℝ^*B*×*D*×*H*×*W*×*C*^, the entire process is divided into two stages. In the first stage, we apply LayerNorm (LN), followed by VSSM to capture long-range dependencies. Additionally, a skip connection is used, as in Equation 5:


$$\begin{array}{l} H^{l}=\text{VSSM}\left(\text{LN}\left(Z^{l}\right)\right)+Z^{l}. \end{array}$$ (5)

In the second stage, a channel attention mechanism is introduced. In this process, a skip connection is also used. This can be expressed as in Equation 6:


$$\begin{array}{l} Z^{l+1}=\text{CA}\left(\text{LN}\left(H^{l}\right)\right)+H^{l}, \end{array}$$ (6)

where CA denotes the channel attention mechanism.

The RVSSM, which incorporates different scanning strategies, will be used to build our network. Further details about the network will be provided in the following sections.

### 3.3 Scanning strategy

**Figure 3:**
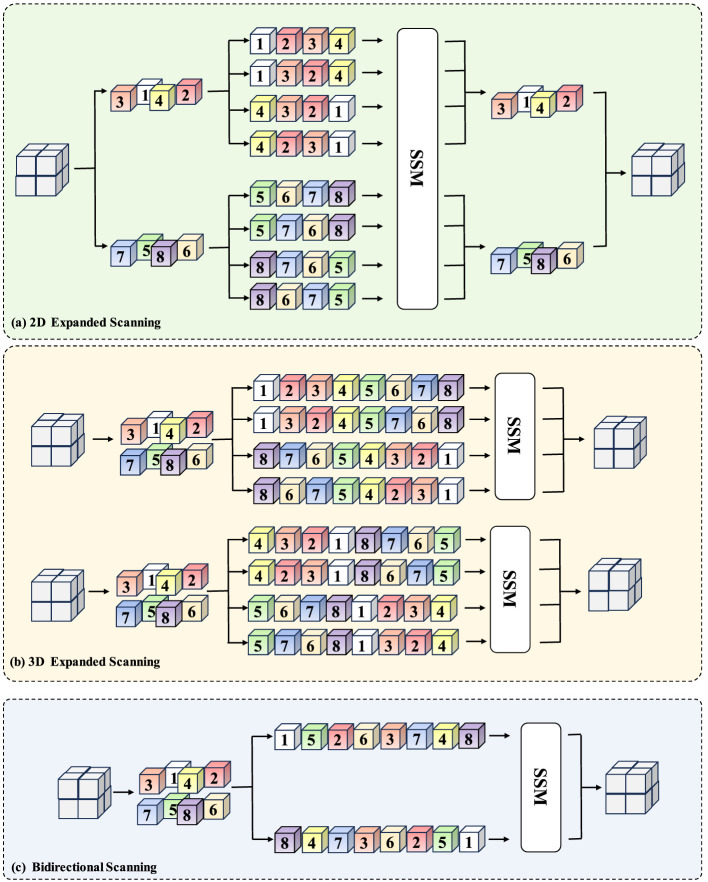
**(a)** The detail of 2D Expanded Scanning. **(b)** The detail of 3D Expanded Scanning. **(c)** The detail of Bidirectional Scanning.

To better adapt to anisotropic volumetric biomedical image structures, we design direction-aware state space scanning strategies at different stages of the network (Figure 3). Given the input feature tensor


$$z\in {\mathbb{R}}^{B\times C\times D\times H\times W},$$

where *B* denotes the batch size, *C* the number of channels, and (*D, H, W*) the spatial dimensions, we reorganize 3D feature maps into ordered sequences along specific spatial directions. For a scanning direction *d*_*k*_, a permutation operator $P_{d_{k}}$ unfolds the feature into a 1D sequence *x*^(*k*)^, which is processed by a State Space Model (SSM):


$$s_{t}^{\left(k\right)}=As_{t-1}^{\left(k\right)}+Bx_{t}^{\left(k\right)},\;y_{t}^{\left(k\right)}=Cs_{t}^{\left(k\right)},$$

where *s*_*t*_ denotes the hidden state and *A, B, C* are learnable parameters. The processed sequence is reshaped back to the 3D domain via $P_{d_{k}}^{-1}$ and aggregated across directions to produce the output feature. This mechanism enables long-range dependency modeling with linear computational complexity.

#### 3.3.1 2D expanded scanning

In shallow layers, we restrict modeling to the *x*-*y* plane to efficiently capture fine-grained intra-slice structures. For a fixed depth index *d*, the slice *z*_:, :, *d*, :, :_ is unfolded along four planar directions


$$D_{2D}=\left\{\to ,\leftarrow ,↓,↑\right\}.$$

Each directional sequence is independently processed by the SSM according to the recurrence above. After sequence processing, the four outputs are reshaped and merged to restore the original 3D tensor structure. This strategy preserves anisotropic characteristics while maintaining computational efficiency at high spatial resolutions.

#### 3.3.2 3D expanded scanning

In deeper layers, scanning is extended to the entire volumetric feature block to integrate global 3D context. The full tensor is unfolded into sequences spanning the domain ℝ^*D*×*H*×*W*^, allowing long-range dependencies across slices to be captured. From a convolutional perspective, the resulting operation can be interpreted as applying a structured global kernel:


$$y_{t}=\overset{L}{\underset{i=0}{\sum }}CA^{\left|t-i\right|}Bx_{i},$$

which aggregates volumetric context while preserving linear complexity. The processed sequences are then reshaped back to the 3D spatial domain.

#### 3.3.3 Bidirectional scanning

To further enhance inter-slice dependency modeling, we introduce bidirectional state updates. For each sequence, forward and backward recursions are computed:


$$s_{t}^{\to }=As_{t-1}^{\to }+Bx_{t},s_{t}^{\leftarrow }=As_{t+1}^{\leftarrow }+Bx_{t},$$

and fused as


$$y_{t}=C\left(s_{t}^{\to }+s_{t}^{\leftarrow }\right).$$

This symmetric formulation allows each voxel to aggregate contextual information from both directions along the scanning axis. Such bidirectional propagation is particularly beneficial for elongated biological structures (e.g., neurons and mitochondria) that extend continuously across slices, improving volumetric consistency and reducing cross-plane discontinuities.

### 3.4 Network architecture

#### 3.4.1 Overview

Our network draws on the design concept of U-Net (Ronneberger et al., 2015) architecture, primarily composed of an encoder, a bottleneck, and a decoder. Specifically, the encoder includes a standard convolutional layer, three SSM-Based anisotropic adaptation module, and three down-sampling layer. The decoder comprises three anisotropic convolutional (AC) module, three up-sampling layer, and a final standard convolutional layer for mask prediction. Additionally, the bottleneck section contains two SSM-Based isotropic adaptation module, one down-sampling layer, one up-sampling layer, and an isotropic convolutional (IC) module designed to support adaptive isotropic feature learning for the decoder. Inspired by the UNETR (Hatamizadeh et al., 2022), encoder cleverly integrates with the CNN-based decoder through multiple skip connections of different resolutions. These skip connections facilitate the recovery of fine-grained details in predictions (Ronneberger et al., 2015; Zhou et al., 2023).

In the following, we present a detailed demonstration of the forward procedure in our network.

#### 3.4.2 Encoder

The input to our network is a 3D patch *X*∈ℝ^*H*×*W*×*D*^ , typically randomly cropped from the original image, where *H*, *W* and *D* denote the height, width and depth of each input, respectively.

##### 3.4.2.1 Conventional convolutional layer

The conventional convolutional layer is responsible for transforming each input *X* into a high-dimensional tensor *X*∈ℝ^*H*×*W*×*D*×*C*^, where *C* denotes the number of channels. Additionally, conventional convolutional layer is capable of extracting shallow features from the input data.

##### 3.4.2.2 SSM-Based anisotropic adaptation (SAA) module

After the conventional convolutional layer, we pass the high-dimensional tensor *X* to the SSM-Based anisotropic adaptation module. The key point behind this is to use RVSSM to scan the x-y plane, as well as the z-axis. At the same time, after scanning the x-y plane and the z-axis using RVSSM, we cascade a convolutional layer and a residual block. For the x-y plane scan, the concatenated convolutional layer and residual block all use a 1 × 3 × 3 anisotropic convolution. After the z-axis scan, a 3 × 1 × 1 anisotropic convolution is applied. Finally, a 1 × 1 × 1 convolution is applied to the output to adjust the dimension of the feature channel, thereby preventing uncontrollable increases in computational complexity. Through this design, the SSM can capture long-range dependencies, while anisotropic convolution can capture local dependencies to compensate for the shortcomings of the former in capturing local dependencies. On the other hand, two different scanning methods and two anisotropic convolution are used to learn features in the x-y plane and z direction respectively, reducing the impact of anisotropic resolution.

**Figure 4:**
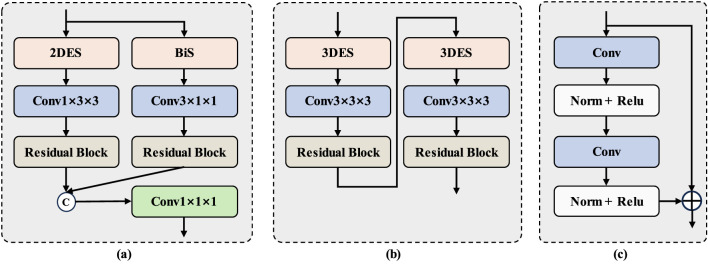
**(a)** SSM-Based Anisotropic Adaptation Module. **(b)** SSM-Based Isotropic Adaptation Module. **(c)** Residual Block. In **(a)**, **(b)**, 2DES represents 2D Extended Scanning, BiS denotes Bidirectional Scanning, and 3DES refers to 3D Extended Scanning. These scanning strategies are integrated with the RVSSM to form the module presented here. For simplicity of expression, we only highlight the scanning strategies in this paper.

Let $f_{l}^{i-1}\in {\mathbb{R}}^{B\times H\times W\times D\times C}$ denote the input to the SSM-Based anisotropic adaptation module, where *B* is the batch size and *C* represents the number of channels. $f_{l}^{i-1}$ is processed through two branches to perform feature learning from the x-y plane and the z-axis, respectively. As shown in Figure 4a, $f_{l}^{i-1}$ enters the left and right branch modules, which are structurally identical but differ in their operations on $f_{l}^{i-1}$. Structurally, the left and right module are identical, but they perform different operations on $f_{l}^{i-1}$. When feature learning is performed in the x-y plane, $f_{l}^{i-1}$ is flattened to $T_{h}^{i}\in {\mathbb{R}}^{\left(BD\right)\times \left(HW\right)\times C}$, after which RVSSM performs extended scanning on it. Upon completion of scanning, extended merging is applied. Finally, $T_{h}^{i}$ is reshaped to $T_{h}^{i}\in {\mathbb{R}}^{B\times H\times W\times D\times C}$ and passed to subsequent anisotropic convolutions for local feature learning. In contrast to learning features in the x-y plane, when performing feature learning along the z-axis, $f_{l}^{i-1}$ is flattened to $T_{w}^{i}\in {\mathbb{R}}^{B\times \left(DHW\right)\times C}$, followed by bidirectional scanning by RVSSM. After scanning, $T_{w}^{i}$ is reshaped back to $T_{w}^{i}\in {\mathbb{R}}^{B\times H\times W\times D\times C}$ and passed to subsequent anisotropic convolution for local feature learning, where a 3 × 1 × 1 anisotropic convolution is applied, as previously described. Finally, the outputs of the two branches are fused to form $T_{c}^{i}\in {\mathbb{R}}^{B\times H\times W\times D\times 2C}$, followed by a 1 × 1 × 1 convolution to adjust the channel number to *C*, resulting in the final output. The computation process can be summarized as in Equation 7.


$$\begin{array}{l} {\bar{T}}_{h}^{i}=RS\left(Conv\left(2DES\left(T_{h}^{i}\right)\right)\right),\\ {\bar{T}}_{w}^{i}=RS\left(Conv\left(BiS\left(T_{h}^{i}\right)\right)\right),\\ T_{c}^{i}=Concat\left({\bar{T}}_{h}^{i},{\bar{T}}_{w}^{i}\right),\\ f_{l}^{i}=Conv\left(T_{c}^{i}\right), \end{array}$$ (7)

where RS denotes residual block, $f_{l}^{i}$ denotes output.

##### 3.4.2.3 Down-sampling layer

**Figure 5:**
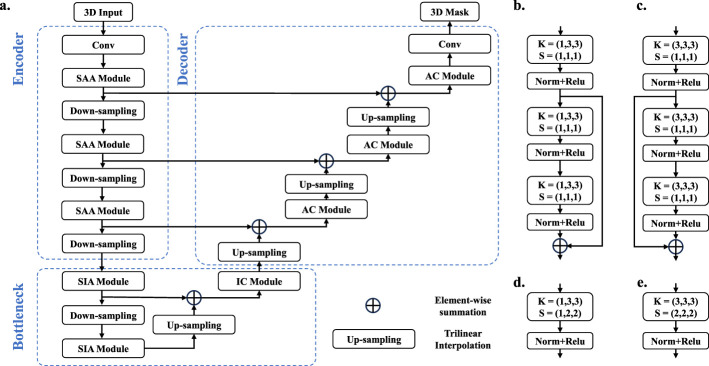
Architecture of network. In **(a)**, we display the overall architecture of network. In **(b)**, we display the anisotropic convolutional (AC) module. In **(c)**, we display the isotropic convolutional (IC) module. In **(d)**, **(e)**, we show how down-sampling is achieved. In **(b–e)**, K refers to the convolutional kernel size, S stands for the stride and Norm denotes the layer normalization strategy.

In the encoder, down-sampling is achieved using simple strided convolutions. As shown in Figure 5d, to address the anisotropy of the down-sampled volume, we set the stride to 2 along two dimensions and 1 along one dimension, thus performing down-sampling primarily in the x-y plane. The detailed parameters are shown in Table 1, this setup helps prevent issues related to limited slice numbers and maximizes feature extraction along the z-axis.

**Table 1:** Configurations of our network on three datasets.

	SNEMI3D	MitoEM-R
Input size	8 × 256 × 256	8 × 256 × 256
DS Str	[1,2,2],[1,2,2],[1,1,1],[2,2,2]	[1,2,2],[1,2,2],[1,1,1],[2,2,2]

DS Str denotes down-sampling stride.

#### 3.4.3 Bottleneck

##### 3.4.3.1 SSM-based isotropic adaptation (SIA) module

In the bottleneck stage, after several down-sampling operations, the volume becomes approximately isotropic. This condition facilitates the application of the SSM-Based isotropic adaptation module. Compared to the SSM-Based isotropic adaptation module, the SSM-Based isotropic adaptation module is more suitable for the processing of isotropic volume features. A SSM-Based isotropic adaptation module consists of two RVSSM, two convolution layer and two residual block. RVSSM is cascaded with a convolution layer and a residual block, and this combination is repeated to form our SSM-Based isotropic adaptation module. It is worth noting that RVSSM employs different 3D selective scanning strategies. Convolution layer and residual block use 3 × 3 × 3 isotropic convolution.

Let $f_{g}^{i-1}\in {\mathbb{R}}^{B\times H\times W\times D\times C}$ represent the input to the SSM-Based isotropic adaptation module, where *B* is the batch size, *C* denotes the number of channels, and *H*, *W*, and *D* represent the spatial dimensions.

At first, $f_{g}^{i-1}$ is flattened into $T_{g}^{i}\in {\mathbb{R}}^{B\times \left(DHW\right)\times C}$, after which it is subjected to an extended scanning operation via the RVSSM. Upon completion of the scanning process, the features are merged back to form an extended representation. Subsequently, $T_{g}^{i}$ is reshaped back into its original dimensions, $T_{g}^{i}\in {\mathbb{R}}^{B\times H\times W\times D\times C}$, and passed to subsequent isotropic convolution for local feature learning. This process is repeated in the second phase, employing a different 3D expanded scanning. The computation process can be summarized as in Equation 8:


$$\begin{array}{c} {\bar{T}}_{g}^{i}=RS\left(Conv\left(3DES\left(T_{g}^{i}\right)\right)\right),\\ f_{g}^{i}=RS\left(Conv\left(3DES\left({\bar{T}}_{g}^{i}\right)\right)\right), \end{array}$$ (8)

where $f_{g}^{i}$ denotes output.

##### 3.4.3.2 Down-sampling layer

In the bottleneck, simple stride convolution is still employed for down-sampling. However, since the down-sampled volume at this stage is approximately isotropic, as illustrated in Figure 5e, the stride is uniformly set to 2 across all dimensions.

#### 3.4.4 Decoder

In the decoder, three anisotropic convolutional modules are constructed using a combination of convolutional operations and a residual block, where a 1 × 3 × 3 anisotropic convolution is used to extract anisotropic features. Compared to the down-sampling layer, trilinear interpolation is used to up-sample low-resolution feature maps to high-resolution ones. These are then fused with the representations of encoder via skip connections to capture fine-grained information. Finally, a convolution layer is applied to generate the final predictions.

### 3.5 Total loss

The standard cross-entropy loss function evaluates the predicted class for each pixel individually and then averages the classification results across all pixels. Since pixel predictions are treated independently and equally, no distinction exists between majority and minority class pixels from the pixel-level perspective. However, in terms of class distribution, there is a significant imbalance, with the number of pixels varying greatly between classes. The pronounced class imbalance poses significant challenges for the model in learning the features of the minority class, resulting in higher false negative predictions for the pixel values and substantially compromising the accuracy of segmentation (Zhang et al., 2023). Pixels belonging to the background or larger objects typically represent the majority class, making cross-entropy loss less effective for imbalanced classification problems. Therefore, we employ Dice loss, defined as in Equation 9:


$$\begin{array}{c} L_{\text{Dice}}=1-\frac{2|A\cap B|}{\left|A|+|B\right|}, \end{array}$$ (9)

where *A* is the set of ground truth pixels, and *B* is the set of predicted pixels. Dice loss is a region-based loss function that measures the mismatch between actual values and predictions across a region. Consequently, the loss and gradient for a single pixel depend not only on its own ground truth label and predicted value but also on the labels and predictions of surrounding pixels, effectively addressing the class imbalance issue in segmentation tasks.

The Dice loss function can be further defined in Equation 10:


$$\begin{array}{c} L_{\text{Dice}}^{\left(2\right)}=1-\frac{2tp+\epsilon }{t+p+\epsilon }, \end{array}$$ (10)

where ϵ is a smoothing factor, *t* represents the ground truth label, and *p* represents the predicted label. The activation function in a binary classification network is formalized in Equation 11:


$$\begin{array}{c} L=\alpha L_{\text{Dice}}+\beta L_{\text{WBCE}}, \end{array}$$ (11)

where *y*∈(0, 1) denotes the output probability of network.

In training, utilizing Dice loss alone may result in instability, especially when misclassifying pixels associated with minor target regions, which can significantly impact the overall loss. When ϵ = 0, the gradient of the Dice loss function is derived in in Equation 12:


$$\begin{array}{c} \frac{dL_{\text{Dice}}^{\left(2\right)}}{dp}=\frac{-2t^{2}}{{\left(t+p\right)}^{2}}, \end{array}$$ (12)

where *t* represents the ground truth label and *p* the predicted value. As (*t, p*) → (0, 0), different conditions yield distinct results. To counter this, we employ a composite loss function that combines a weighted binary cross-entropy (WBCE) loss with the Dice loss, optimized as in in Equation 13:


$$\begin{array}{c} L=\alpha L_{\text{Dice}}+\beta L_{\text{WBCE}}, \end{array}$$ (13)

where α and β are weighting coefficients. The WBCE loss function *L*_WBCE_ is specified by Equation 14:


$$\begin{array}{c} L_{\text{WBCE}}\left(Y_{i},G_{i}\right)=\frac{1}{DHW}W_{i}L_{\text{BCE}}\left(Y_{i},G_{i}\right), \end{array}$$ (14)

where *Y*_*i*_ and *G*_*i*_ indicate the predicted and target values for the i-th block, and *D*, *H* and *W* denote the height, and width of the block. The weighting term *W*_*i*_ is defined by Equation 15:


$$W_{i}=\{\begin{array}{ll} G_{i}+\frac{W_{f}}{1-W_{f}}(1-G_{i}) & \text{if }W_{f}>0.5\\ \frac{W_{f}}{1-W_{f}}G_{i}+(1-G_{i}) & \text{if }W_{f}\leq 0.5, \end{array}$$ (15)

where *W*_*f*_ denotes the foreground proportion, calculated as $W_{f}=\frac{\sum Y_{i}}{DHW}$.

For the SNEMI3D dataset, the comprehensive loss function is expressed as Equation 16:


$$\begin{array}{c} L_{\text{all}}=L\left(Y_{A},G_{A}\right), \end{array}$$ (16)

where *Y*_*A*_ and *G*_*A*_ represent the predicted and actual affinity maps, respectively.

For the MitoEM-R dataset, the comprehensive loss function is defined as Equation 17:


$$\begin{array}{c} L_{\text{all}}=L\left(Y_{M},G_{M}\right)+L\left(Y_{B},G_{B}\right), \end{array}$$ (17)

where *Y*_*M*_ and *Y*_*B*_ are the predicted semantic mask and instance boundary maps, and *G*_*M*_ and *G*_*B*_ are their respective ground truth.

Through a carefully weighted combination, this composite loss function facilitates training stability while enhancing classification accuracy under conditions of class imbalance.

## 4 Experiments

### 4.1 Datasets

#### 4.1.1 SNEMI3D

The SNEMI3D dataset (Lee et al., 2017) consists of images collected from the visual cortex of mice, generated using serial sectioning scanning electron microscopy. The images are produced by slicing tissue along the z-axis, with a resolution of 6 × 6 × 30 nm/voxel, covering an approximate micro-cube of 6 × 6 × 3 micrometers. The dataset comprises two image stacks designated for training and testing, with each stack containing 100 slices of 1024 × 1024 pixels. Only the training image stack is accompanied by labels. To evaluate our method, we divided the training stack into the first 80 slices for training and the remaining 20 slices for validation.

#### 4.1.2 MitoEM-R

The MitoEM-R dataset (Wei et al., 2020) originates from the layer II/III of the primary cortex of adult rats, with images obtained using multibeam scanning electron microscopy for a tissue volume of 30 μ*m*^3^. The imaging resolution for the tissue is 8 × 8 × 30nm^3^. The dataset consists of three components: the training set 400 × 4096 × 4096, the validation set 100 × 4096 × 4096, and the test set 500 × 4096 × 4096.

### 4.2 Evaluation metrics

#### 4.2.1 SNEMI3D

We utilize two widely recognized metrics for quantitative evaluation of neuron instance segmentation: Variation of Information (*VOI*) (Meilă, 2003) and Adapted Rand Error(*ARAND*). The VOI metric assesses the discrepancy between the segmentation results and the ground truth, comprising two components: *VOI*_s_, which quantifies over-merging, and *VOI*_M_, which measures over-segmentation errors. The ARAND metric, an extension of the Rand Index, is adjusted to account for the uneven distribution of object sizes in electron microscopy (EM) image segmentation (Liu et al., 2024b; Rand, 1971). It measures the degree of consistency between the ground truth and the segmentation output. It is worth emphasizing that lower values of both metrics indicate superior segmentation performance.

#### 4.2.2 MitoEM-R

In this paper, we use AP-75 as the evaluation metric, which requires that the predicted instance and the ground truth instance have at least 75% IoU intersection to be considered TP (following Li et al., 2021), otherwise the ground truth instance is considered a FN and the prediction a FP. Furthermore, based on the voxel size of the instance, the predicted instance and the ground instance can be further divided into small objects(< 5*k* voxels), medium objects and large objects(>30*k* voxels) (Franco-Barranco et al., 2023). Therefore, we can finally get the evaluation results of AP-75 on small objects, medium objects, and large objects.

### 4.3 Implementation details

All experiments were performed using the AdamW optimizer (Loshchilov and Hutter, 2017), with hyperparameters configured as β_1_ = 0.9 and β_2_ = 0.999, a learning rate of 1 × 10^−4^, and a batch size of 6. The training process was conducted 200,000 iterations on six NVIDIA RTX 3090 GPUs. During the training stage, we apply a series of augmentation techniques (Lin et al., 2021) in the specified order: rotation, scaling, flipping, elastic deformation, missing parts, missing slices, motion blur, and dislocation. In the inference stage, we use a Gaussian function to combine overlapping sliding window volumes in order to mitigate boundary artifacts. For the SNEMI3D dataset, two different post-processing algorithms(waterz Funke et al., 2018 and LMC Beier et al., 2017) are used to process affinity maps to generate neuron instances. In contrast, for the MitoEM-R dataset, we employ a marker-controlled watershed algorithm (Wei et al., 2020) to process binary masks and instance contours to generate instances. It is worth emphasizing that uniform post-processing settings were employed across all experiments to maintain consistency. These settings were aligned with those of existing baseline methods, ensuring that the outcomes and conclusions derived from our approach remain unaffected by variations introduced during the post-processing stage.

### 4.4 Comparison experiments

To validate the effectiveness of our proposed network, we perform a comprehensive comparison with a set of state-of-the-art algorithms, which include successful methods for 3D biomedical data segmentation, such as CNN-based UNet3D (Çiçek et al., 2016), and Transformer-based architectures like SwinUNETR (Liu et al., 2021) and UNETR (Hatamizadeh et al., 2022), as well as the SegMamba (Xing et al., 2024) built on the Mamba architecture. In addition, we examine methods specific to certain datasets. For the SNEMI3D dataset, we evaluated Superhuman (Lee et al., 2017), MALA (Funke et al., 2018) and LSD (Sheridan et al., 2023), while for the MitoEM-R dataset, comparisons were made with U3D-BC (Wei et al., 2020) and Res-UNet-R (Li et al., 2022).

#### 4.4.1 Neuron segmentation (SNEMI3D)

Table 2 presents the experimental results of all models on the neuronal segmentation task. Our method outperforms state-of-the-art approaches in most cases. Using the waterz post-processing method, our approach demonstrates superior performance compared to CNN-based, transformer-based, and Mamba-based methods. Swin-u and UNETR may have strong capabilities in long sequence modeling due to the use of Transformer, but their ability to extract local features is insufficient, resulting in split erros and merge errors. MALA and Superhuman use convolution and have a strong advantage in local feature extraction, but when faced with large targets such as neurons, they show insufficient global modeling capabilities and have many merge errors. Although LSD adopts auxiliary representations and a specific training strategy, its underlying network architecture remains a standard 3D U-Net. As a result, it is limited in global context modeling, and its performance is slightly inferior to that of our proposed network. While LPEA-HIS (Huang et al., 2022) emphasizes learning pixel-level embedding affinities, it has limited ability to capture long-range dependencies, which results in performance that is slightly lower than that of our proposed method. Although SegMamba uses Mamba as the backbone, it does not design modules specifically for the characteristics of electron microscope characteristics, and the segmentation effect is not particularly ideal. Notably, our method achieved a significant improvement in the VOI metric, surpassing the second-best method by 17.6%.

**Table 2:** Comparison of segmentation results on SNEMI3D dataset.

Post.	Method	VOI_S_↓	VOI_M_↓	VOI↓	ARAND↓
Waterz	3D U-Net	2.7665	1.8383	4.6048	0.7918
	Swin-U	0.4068	0.2801	0.6869	0.0597
	UNETR	0.5768	0.3888	0.9656	0.1298
	Tiny-UNETR	0.5187	0.3125	0.8312	0.1135
	SegMamba	0.4648	0.1864	0.6512	0.0503
	Superhuman	0.4247	0.1529	0.5776	**0.0432**
	MALA	0.5102	0.1944	0.7046	0.0542
	LSD	0.3325	0.1628	0.4953	0.0565
	LPEA-HIS	0.3438	0.2245	0.5683	0.0573
	**Ours**	**0.3235**	**0.1524**	**0.4759**	0.0533
LMC	3D U-Net	1.7641	1.7082	3.4723	0.5646
	Swin-U	0.6527	0.1637	0.8164	0.1165
	UNETR	0.8379	0.1857	1.0236	0.1531
	Tiny-UNETR	0.7911	0.1725	0.9636	0.1435
	SegMamba	0.6745	0.1509	0.8254	**0.1078**
	Superhuman	0.9575	0.1409	1.0984	0.1501
	MALA	0.7652	0.1616	0.9268	0.1203
	LSD	0.7035	0.1228	0.8263	0.1237
	LPEA-HIS	0.6936	0.1357	0.8293	0.1436
	**Ours**	0.6756	**0.1359**	**0.8115**	0.1128

‘Post.' denotes the post-processing algorithms. Best results in bold, second-best underlined.

**Figure 6:**
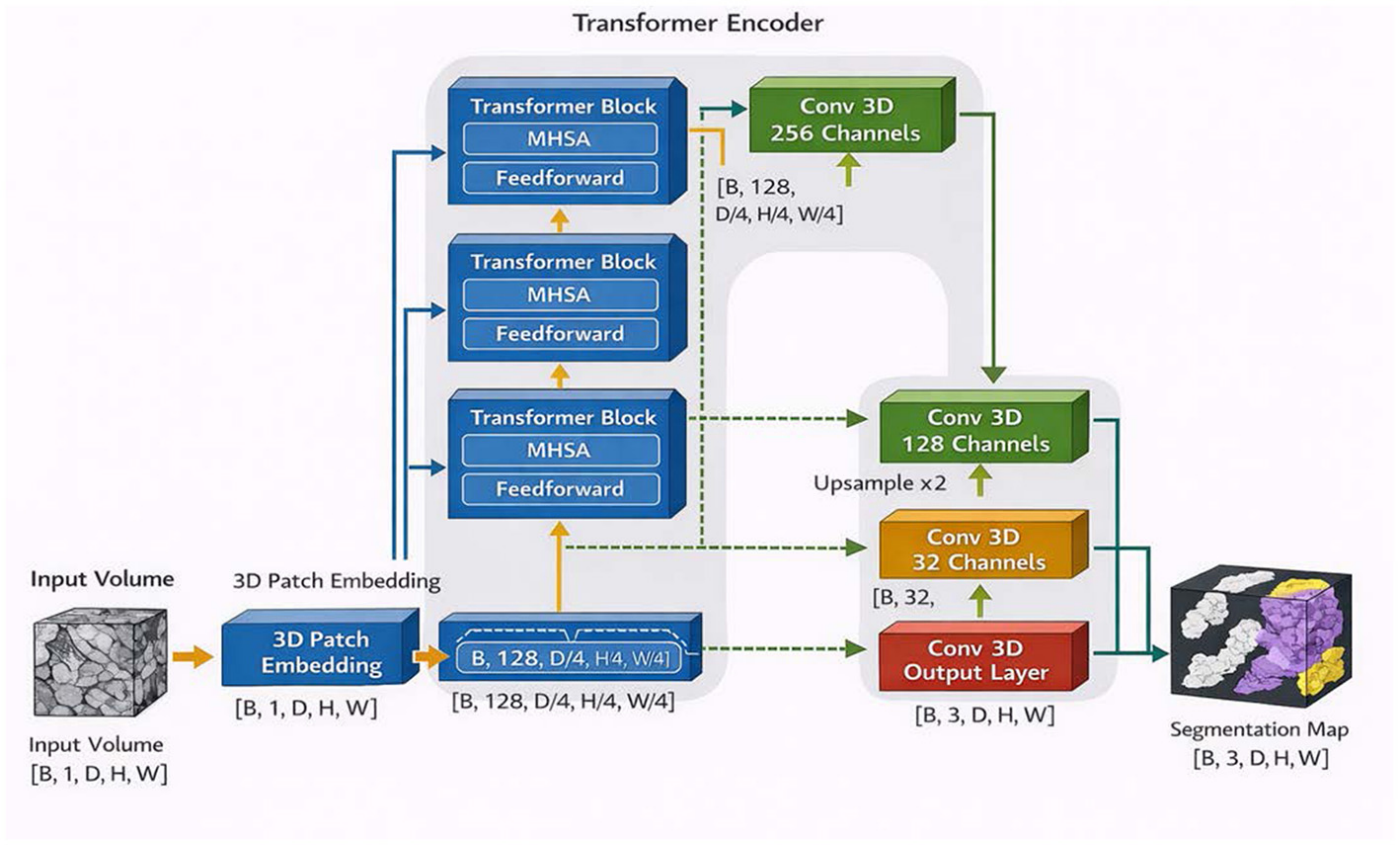
Tiny 3D UNETR architecture.

To demonstrate that the model performance is primarily related to the architectural design rather than being negatively correlated with the number of parameters, we adopted the network architecture shown in Figure 6, which contains approximately 3 million parameters. All experiments were conducted on the same dataset, using identical data augmentation strategies and evaluation metrics to ensure a fair comparison.

According to the experimental results, we observe that even with a substantial reduction in model parameters—specifically when employing the lightweight UNETR architecture—the segmentation performance does not show a significant improvement. As shown in Table 4, reducing the number of model parameters does not necessarily lead to better performance. These results suggest that model performance is more closely related to the architectural design rather than simply the parameter scale.

#### 4.4.2 Mitochondria segmentation(MitoEM-R)

Table 3 summarizes the experimental results on the MitoEM dataset. Among CNN-based, Transformer-based, and Mamba-based methods, the best performing model is the Mamba-based SegMamba. For the MitoEM-R dataset specifically, the top-performing model is Res-UNet-R. In comparison, our method demonstrates superior performance in the AP-75 metric, outperforming both SegMamba and Res-UNet-R. This further validates the advantage of our approach over previous methods.

**Table 3:** Comparison of segmentation results on MitoEM-R dataset (AP-75).

Method	Small	Med	Large	ALL
3D U-Net	0.023	0.752	0.834	0.787
Swin-U	0.136	0.831	0.840	0.815
UNETR	0.062	0.751	0.803	0.762
SegMamba	0.225	0.835	0.870	0.838
U3D-BC	0.229	0.829	0.881	0.837
Res-UNet-R	0.291	0.833	0.900	0.885
**Ours**	**0.369**	**0.842**	**0.925**	**0.891**

Bold values indicate the optimal results.

#### 4.4.3 Model complexity comparison

To assess the computational advantages of the proposed method, we perform experiments on the SNEMI3D dataset. In Table 4, we compare the complexity of different models. Compared to convolution-based U-Net3D, transformer-based models such as SwinUNETR and UNETR, and Mamba-based SegMamba, our model demonstrates smaller model size and computational complexity. Furthermore, compared to Superhuman and MALA, which are specifically designed for neuronal segmentation, our model achieves the smallest computational complexity while maintaining a model size comparable to that of Superhuman.During inference, a unified configuration was adopted for all methods. The input patch size was set to 8 × 256 × 256 with a sliding-window stride of 4 × 128 × 128. These parameters were kept identical across all models, as they have a significant impact on the measured wall-clock inference time. Under this standardized evaluation protocol, our method achieves the fastest inference speed compared with other competing models.

**Table 4:** Quantitative comparison of model complexity on SNEMI3D dataset.

Method	VOI	#Params (M)	FLOPs (GMAC)	Latency (s)
3D U-Net	4.6048	12.95	220.36	193.81
Swin-U	0.6869	15.70	98.97	362.54
UNETR	0.9656	92.38	86.02	204.10
SegMamba	0.6512	67.36	388.43	449.04
Superhuman	0.5776	**1.48**	523.86	113.83
MALA	0.7049	84.02	207.09	160.94
**Ours**	**0.4759**	3.05	**41.91**	**41.98**

VOI results are obtained by the waterz post-processing. Bold values indicate the optimal results.

### 4.5 Ablation study

We conduct ablation study on the MitoEM-R validation set using AP-75 as the evaluation metric. As shown in Table 5, we perform the ablation study on the key components of our proposed method, including the SAA and SIA module. The decoder of the baseline network in the table consists of three anisotropic convolution module and three down-sampling layer, with the bottleneck consisting of three isotropic convolution module and one down-sampling layer, followed by an up-sampling layer. The dencoder is the same as in our network. “+ SAA” indicates the replacement of the three anisotropic convolution module in the baseline decoder with the SAA module, while “+ SIA” indicates the replacement of the two isotropic convolution module in the down-sampling process of the bottleneck with the SIA module.

**Table 5:** The results of ablation study on MitoEM-R validation set (AP-75).

Models	Small	Med	Large	ALL
Baseline	0.342	0.854	0.844	0.834
Baseline + SAA	0.317	0.847	0.859	0.846
Baseline + SIA	0.365	0.873	0.890	0.869
Baseline + SAA + SIA	**0.451**	**0.874**	**0.905**	**0.890**

Bold values indicate the optimal results.

From Table 5, it can be seen that replacing the anisotropic convolution module with the SAA improves the model performance by 1.4%, while replacing the anisotropic convolution module with SIA results in a 4.2% improvement. This phenomenon demonstrates the effectiveness of our proposed method. When both the anisotropic convolution module and isotropic convolution module are replaced, the model performance improves by 6.7%. This suggests that providing a sufficiently large receptive field is beneficial for segmentation tasks.

To validate the rationality of our architectural design, we conducted an ablation study by evaluating different combinations of the proposed modules, including variants that use only 2D Expanded Scanning, only 3D Expanded Scanning, and only Bidirectional Scanning across all layers. All experiments were performed on the same dataset, using identical data augmentation strategies and evaluation metrics to ensure a fair comparison.

As shown in Table 6, the model achieves the worst performance when only Bidirectional Scanning is employed. This may be attributed to the fact that bidirectional scanning primarily focuses on the z-axis information and fails to effectively capture structural information in the xy plane. When only 2D Expanded Scanning is applied, the performance improves to some extent. This is likely because the model emphasizes the xy plane, which contains richer structural information. However, due to the lack of sufficient modeling along the z-axis, the performance remains limited.

**Table 6:** The results of on MitoEM-R validation set (AP-75).

Models	Small	Med	Large	ALL
All 2D (SAA)	0.293	0.812	0.735	0.799
All 3D (SIA)	0.333	0.836	0.834	0.821
All Bidirectional	0.123	0.788	0.743	0.712
ours	**0.451**	**0.874**	**0.905**	**0.890**

Bold values indicate the optimal results.

When only 3D Expanded Scanning is used, the performance becomes the second best among all variants, slightly inferior to our proposed hybrid strategy. This is likely because 3D scanning can jointly model both xy and z directions, but it does not explicitly account for the anisotropic characteristics of image volumes. As a result, its performance is slightly lower than that of our method.

### 4.6 Visualization of segmentation results

**Figure 7:**
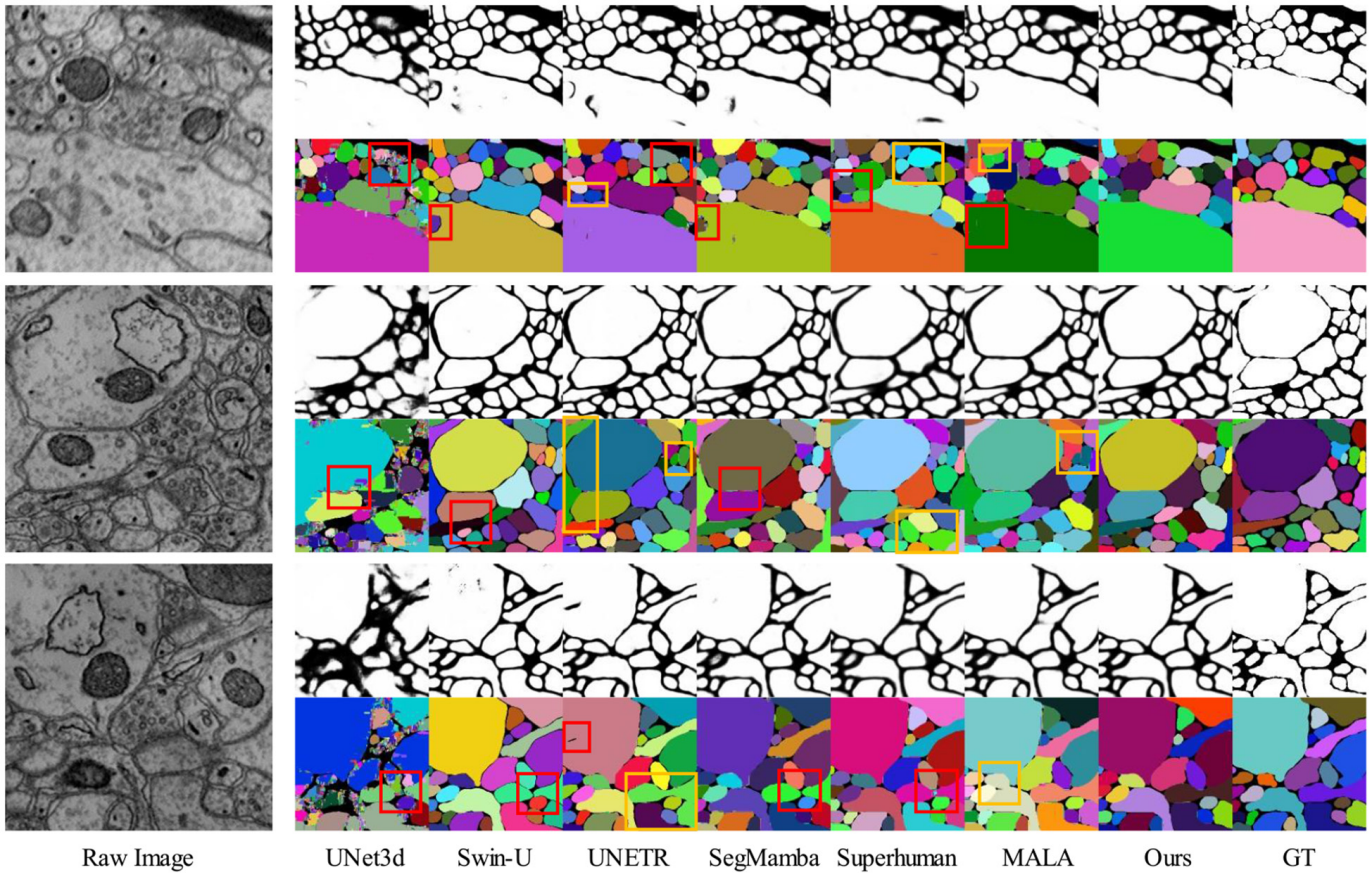
The 2D visual results on SNEMI3D dataset. We display three sets of images. Within each set, the leftmost row shows the original image, the first row shows the affinity map, and the second row shows the instance segmentation results corresponding to the affinity map. Red and orange boxes indicate split and merge errors, respectively.

**Figure 8:**
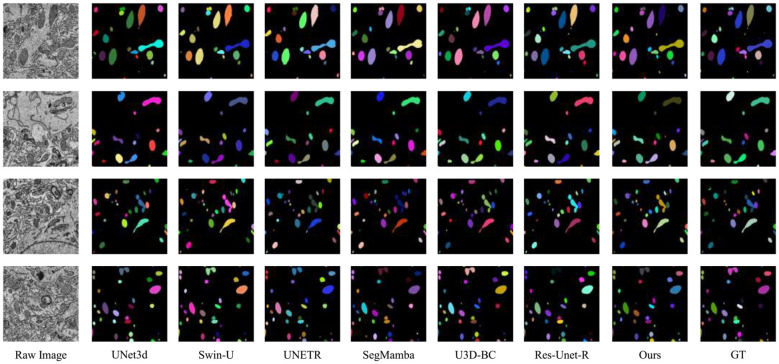
The 2D visual results on MitoEM-R dataset. We display four sets of images.

We present the results of the 2D visual comparison in Figures 7, 8. As shown in Figure 7, our proposed method outperforms other baseline methods in predicting affinity maps with higher fidelity, significantly reducing split and merge errors and more accurately preserving the neuronal structure. Figure 8 shows that our method provides mitochondrial predictions that are closer to the truth of the ground, highlighting the superiority of our approach. These results validate the effectiveness of our model.

## 5 Conclusion

In this paper, we propose a state-space model (i.e., Mamba)-based approach for segmentation of brain structure from 3D EM image. Specifically, we introduce an adaptive scanning method targeting the anisotropic voxels of 3D EM image. Based on our proposed scanning method, we design SSM-based anisotropic adaptation module and SSM-based isotropic adaptation module for feature learning. In addition, the convolutional layers and the SSM-based anisotropic adaptation module and the SSM-based isotropic adaptation module are interwoven to enhance the learning ability of multi-scale features. Extensive experiments show that our method achieves superior segmentation performance with extremely low memory compared to previous methods.

## Data Availability

Publicly available datasets were analyzed in this study. This data can be found here: The datasets analyzed in this study are publicly available at: SNEMI3D https://snemi3d.grand-challenge.org/; MitoEM-R https://mitoem.grand-challenge.org/.
